# Weekly Intra-Amniotic IGF-1 Treatment Increases Growth of Growth-Restricted Ovine Fetuses and Up-Regulates Placental Amino Acid Transporters

**DOI:** 10.1371/journal.pone.0037899

**Published:** 2012-05-22

**Authors:** Jibran A. Wali, Hendrina A. de Boo, José G. B. Derraik, Hui Hui Phua, Mark H. Oliver, Frank H. Bloomfield, Jane E. Harding

**Affiliations:** Liggins Institute, University of Auckland, Auckland, New Zealand; University of Giessen Lung Center, Germany

## Abstract

Frequent treatment of the growth-restricted (IUGR) ovine fetus with intra-amniotic IGF-1 increases fetal growth. We aimed to determine whether increased growth was maintained with an extended dosing interval and to examine possible mechanisms. Pregnant ewes were allocated to three groups: Control, and two IUGR groups (induced by placental embolization) treated with weekly intra-amniotic injections of either saline (IUGR) or 360 µg IGF-1 (IGF1). IUGR fetuses were hypoxic, hyperuremic, hypoglycemic, and grew more slowly than controls. Placental glucose uptake and *SLC2A1* (*GLUT2*) mRNA levels decreased in IUGR fetuses, but *SLC2A3* (*GLUT3*) and *SLC2A4* (*GLUT4*) levels were unaffected. IGF-1 treatment increased fetal growth rate, did not alter uterine blood flow or placental glucose uptake, and increased placental *SLC2A1* and *SLC2A4* (but not *SLC2A3*) mRNA levels compared with saline-treated IUGR animals. Following IGF-1 treatment, placental mRNA levels of isoforms of the system A, y^+^, and L amino acid transporters increased 1.3 to 5.0 fold, while the ratio of phosphorylated-mTOR to total mTOR also tended to increase. Weekly intra-amniotic IGF-1 treatment provides a promising avenue for intra-uterine treatment of IUGR babies, and may act via increased fetal substrate supply, up-regulating placental transporters for neutral, cationic, and branched-chain amino acids, possibly via increased activation of the mTOR pathway.

## Introduction

Intrauterine growth restriction (IUGR) is associated with increased perinatal morbidity and mortality [1] and with increased risk of adult diseases such as diabetes, hypertension, and coronary artery disease [2]. Impaired growth may persist during childhood despite optimum nutrition [3].

Although a poorly growing fetus can be relatively easily identified by obstetric ultrasound, the therapeutic options are limited. Thus, sustained poor growth *in utero* frequently results in the fetus being delivered [4], with the attendant morbidity and mortality of preterm birth. Further, preterm birth is itself associated with hypertension [5], diabetes and insulin resistance [6], ischemic heart disease and stroke [7] in later life.

It is unclear whether intervention early in postnatal life can ameliorate these increased risks. For example, accelerated postnatal growth may increase the long-term risks associated with reduced size at birth [8], [9]. Therefore, attempting to reverse IUGR *in utero* may represent the optimum approach [10].

In developed nations, IUGR typically is caused by placental insufficiency, resulting in a reduced fetal nutrient supply [10]. Thus, clinical attempts to improve fetal growth by maternal supplementation with protein [11] or oxygen [12] were unsuccessful. Attempts to increase placental blood flow with sildenafil citrate in IUGR ovine pregnancies resulted in adverse outcome [13], and supplementation of pregnant women carrying severely IUGR fetuses with L-arginine (a nitric oxide precursor) had no effect on fetal growth [14]. Therefore, a treatment that bypasses the placenta may provide the most promising approach.

Insulin-like growth factor-1 (IGF-1) is the primary endocrine regulator of fetal growth in late gestation. Birth weight is positively associated with IGF-1 concentrations in umbilical cord blood, and circulating fetal IGF-1 concentrations are lower in IUGR pregnancies [15]. Acute high-dose intravenous IGF-1 infusion has anabolic effects on fetal sheep, stimulating substrate uptake and inhibiting protein breakdown [16]. However, while continuous vascular access to the fetus is not practicable in IUGR treatment, the amniotic fluid compartment is routinely accessed in clinical practice.

IGF-1 administered into the amniotic fluid in sheep is swallowed and taken up across the fetal gut to circulate intact in the fetus [17]. Further, administration of low dose intra-amniotic IGF-1 thrice weekly increases growth of ovine fetuses with IUGR induced by placental embolization [18]. However, for intra-amniotic IGF-1 treatment to be clinically useful, a less frequent administration is required. In addition, circulating fetal concentrations of IGF-1 following treatment were either unaltered [18] or actually decreased [19], with down-regulation of fetal hepatic *igf1* mRNA levels [20]. These data suggest that the increased fetal growth after intra-amniotic IGF-1 is unlikely to be mediated directly by circulating IGF-1.

One possible mechanism is via effects on placental nutrient transport. Glucose is transported across the placenta by facilitated diffusion, mediated by glucose transporters (members of the SLC2A family). SLC2A1 and SLC2A3 are the major glucose transporter isoforms in the placenta of ruminants and rodents [21], with SLC2A4 also described in humans and rats [22], [23]. In contrast, amino acids are transported across the placenta by active transport mediated by numerous different amino acid transporters, many of which have several isoforms [24]. Recent studies have suggested that changes in placental amino acid transport, possibly mediated by altered activity of mammalian target of rapamycin (mTOR), precede growth restriction and may therefore directly contribute to decreased fetal growth [25]. IGF-1 is known to alter activity and/or expression of nutrient transporters in different placental preparations [26], [27].

This study aimed to determine whether intra-amniotic IGF-1 administration only once a week would still enhance growth of IUGR fetuses. We also aimed to identify possible mechanisms of action by examining the effects of treatment on placental nutrient transporters and mTOR, and on placental and fetal nutrient uptake.

## Materials and Methods

### Ethics statement

Experiments were approved by the University of Auckland Animal Ethics Committee (approval number AEC/04/2004/R369).

**Table 1:** Primer and probe sequences and characteristics.

Gene and Genbank (Accession ID)	Primers and Probes	Sequences	Melting Temperature (°C)	Location on Gene Sequence (base pairs)
***SLC2A1***	Forward	5′-TCC CAG TGG CCC AAG GA-3′	60	1202–1218
(U89029)	Reverse	5′-GAT CTA TCA GTT TGA GAG TCT CAT CCA-3′	58	1239–1265
	Probe	6FAM-TCA GAG CGC AGG CAG-MGBNFQ		1223–1237
***SLC2A3***	Forward	5′-TGC CTT ATG GGA TTC TGC AAA-3′	59	431–451
(NM_001009770)	Reverse	5′-TCA GTC GGC CCA AAA TCA G-3′	58	473–491
	Probe	6FAM-AGC AGA GTC AGT TGA AAT-MGBNFQ		454–471
***SLC2A4***	Forward	5′-AAC CCA GCA CAG AAC TGG AGT AC-3′	59	71–93
(AB005283)	Reverse	5′-CCT GTG TGG ACC CTC AGT CA-3′	58	110–130
	Probe	5′-AGGGCCGGATGAGA-3′		96–109
***SLC38A4***	Forward	5′-TTG TTT ACA CTG AAG GAG CTT GTG T-3′	59	211–235
(XM_605868)	Reverse	5′-CAG TTC CAT GGG ATC CAT TTG-3′	58	260–280
	Probe	6FAM-CAG CGC TTT CTT GTC C-MGBNFQ		238–253
***SLC7A1***	Forward	5′-CCT AGC GCT CCT GGT CAT CA-3′	60	271–290
(AF212146)	Reverse	5′-GGC GTC CTT GCC AAG TAC A-3′	58	307–325
	Probe	6FAM-CCT TCT GCA TGG CAG C-MGBNFQ		291–306
***SLC7A5***	Forward	5′-CTC CAT CCT CTC CAT GAT CCA-3′	58	125–145
(AY162432)	Reverse	5′-TGT GAA CAC CAG TGA GGG TAC AG-3′	59	163–185
	Probe	6FAM-CCG CGG CTG CTG A-MGBNFQ		147–159
***SLC7A8***	Forward	5′-GTC AGA GCC CGT GGT GTG T-3′	58	205–223
(AY162433)	Reverse	5′-TGC CAG TAA ATA CCC AGG AAG TAG A-3′	59	261–285
	Probe	6FAM-CCA TCA TGG TGA CAG GG-MGBNFQ		237–253
**mTOR**	Forward	5′-CTG CAC GTC AGC ACC ATC A-3′	59	3772–3790
(XM_001788228)	Reverse	5′-AGC CAT TCC AAC CAA TCA TCT T-3′	58	3830–3851
	Probe	6FAM-CCT CCA AAA GGC C-MGBNFQ		3792–3804
**Cyclophilin A**	Forward	5′- GTA CTG GTG GCA AGT CCA TCT -3′		105–125
(AY251270.1)	Reverse	5′- CAG GAC CTG TAT GCT TCA GAA TGA -3′		153–176
	Probe	6FAM- ATG GCG AGA AAT TTG-MGBNFQ		126–140
**Beta actin**	Forward	5′- ACC AGT TCG CCA TGG ATG ATG -3′		76–96
(NM_001009784.1)	Reverse	5′- CCG GAG CCG TTG TCA AC -3′		113–128
	Probe	6FAM- ACG AGC GCA GCA ATA T –MGBNFQ		97–112
**GAPDH**	Forward	5′- GGG CTG CTT TTA ATA CTG GCA AA -3′		91–114
(NM_001190390)	Reverse	5′- CAT GTA GAC CAT GTA GTG AAG GTC AA -3′		146–171
	Probe	6FAM- CAT CGT TGC CAT CAA TG –MGBNFQ		120–136
**YWHAZ**	Forward	5′- GAG GGT CGT CTC CAG TAT TGA G -3′		25–46
(AY970970.1)	Reverse	5′- TTC TCG AGC CAT CTG CTG TTT T -3′		70–91
	Probe	6FAM- CAG CAC CTT CCG TCT TT –MGBNFQ		49–65
**HPRT1**	Forward	5′- AGG TGT TTA TTC CTC ATG GAC TAA TTA TGG -3′		1–30
(EF078978)	Reverse	5′- CAC CCA TCT CCT TCA TCA CAT CTC -3′		52–75
	Probe	6FAM- ACA GGA CCG AAC GAC TG –MGBNFQ		31–47
**RPL19**	Forward	5′-CAA AAA CAA GCG GAT TCT CAT G -3′		340–361
(AY158223.1)	Reverse	5′- GCT TCT TGC GAG CCT TGT CT -3′		385–404
	Probe	6FAM- AAC ATA TCC ACA AGC TGA A –MGBNFQ		363–381
**18S**	Eukaryotic 18S rRNA endogenous VIC/MGB Probe and Primers (Applied Biosystems, Foster City, CA, USA)			

FAM / VIC: fluorescent reporter dyes bound to the TaqMan probe. MGBNFQ  =  molecular-groove binding non-fluorescence quencher.

### Animals

Romney ewes carrying singleton fetuses were acclimatized to indoor conditions, housed in individual cages with free access to water and pelleted food. Ewes were randomized to one of three experimental groups: Control, and two groups of embolized fetuses subsequently treated with saline (IUGR) or IGF-1 (IGF1).

Ewes were fasted overnight before surgery under halothane general anesthesia at 98 days gestational age (dGA) (term  = 147 dGA). Catheters were placed in both fetal femoral arteries (FA) and veins (FV) via the tarsal vessels, and the common umbilical vein (UV) [28]. Two amniotic catheters were inserted into the amniotic sac. Growth catheters were inserted around each side of the fetal chest from the sternum to the spine to measure changes in fetal girth [29]. Catheters were also placed in the maternal femoral artery (MA) and vein, the carotid artery and jugular vein and the utero-ovarian vein (UOV) draining the pregnant horn. In animals randomized to the IUGR groups, catheters were inserted into both uterine arteries [30].

Control animals received no embolization and no treatment. In embolized groups, IUGR was induced between 103 and 109 dGA by utero-placental embolization with 20–50 µm polystyrene microspheres (Superose 12, Pharmacia Biotech, Uppasala, Sweden) as described previously [19]. IUGR fetuses received intra-amniotic injections on 110, 117 and 124 dGA of either 360 µg (100 µg/mL) IGF-1 (Genentech Inc, San Francisco, CA, USA) (IGF1 group) or an equivalent volume of sterile saline (IUGR group).

### Sampling

Blood and amniotic fluid samples from FA, MA, UV, UOV and amniotic catheters were taken twice-weekly in the morning before feeding, and before intra-amniotic injection. Samples were placed on ice prior to centrifugation, and the supernatants stored at −80°C until assay. Further aliquots of whole blood were immediately frozen for antipyrine assay and radiotracer counting. For blood gas analysis (Chiron M845 blood gas analyser; Chiron Corp., Emeryville, USA), separate blood samples were collected in heparinized syringes on ice, and analysed within 15 min.

At 120 dGA, the steady-state uterine and fetal uptakes of glucose, oxygen and lactate, and the placental clearance of non-metabolizable tracer analogues of glucose (3-O-[methyl^3^H]glucose) and urea ([^14^C]urea) were assessed using the Fick principle. A tracer solution comprising 160 mg antipyrine, 500 µCi 3-O-[Me^3^H]glucose and 250 µCi [^14^C]urea in 20 ml saline was infused into a fetal vein for 3.5 h at 3 ml/h following a 4 ml bolus. Under these conditions antipyrine reaches steady state after 90 min, and tracers after 2.5 h [30]. From 2.5 h after the start of infusions, five sets of blood samples, consisting of blood withdrawn simultaneously from the FA, UV, MA and UOV, were collected at 15 min intervals.

At 131 dGA ewes were killed with an overdose of pentobarbitone. The fetus was dried, weighed and measured. The uterus, placenta and fetal organs were dissected and weighed. As soon as the fetus was removed from the uterus, three type B placentomes were randomly selected and removed, weighed, and snap frozen in liquid nitrogen and stored at −80°C until analysis.

### Hormone and metabolite assays

Glucose, lactate, and urea concentrations were measured on an autoanalyser (Hitachi 902, Tokyo, Japan) using commercial kits (glucose and urea, Roche, Mannheim, Germany; lactate, Randox Laboratories Ltd, Ardmore, UK). IGF-1 was measured by radioimmunoassay [31]. Progesterone and cortisol concentrations were measured using mass spectrometry [32].

Antipyrine [33] and amino acid concentrations [34] were measured by HPLC as described previously, but with a Phenomenex Luna column (Luna 3 μm C18(2) 100 Å), dimensions 250×4.6 mm (Phenomenex, Torrance, USA) for amino acid analyses. 3-O-[Me^3^H]glucose and [^14^C]urea specific activities were measured in duplicate as described previously [35].

### Real time PCR

Total RNA was extracted using TRIZOL® (Invitrogen, Carlsbad, CA, USA). RNA concentration was measured using a NanoDrop ND-1000 spectrophotometer running 3.1.2 NanoDrop software (BioLab Ltd, Auckland, New Zealand). Absorbance ratios used were 260/280 and 260/230, with ratios ≥1.9 considered acceptable purity. First-strand cDNA was synthesized using Superscript III first strand synthesis system (Invitrogen). Total RNA (5 µg) samples were treated with RNase-free DNase I (Invitrogen) before reverse transcriptase polymerase chain reaction (RT-PCR) to eliminate potential genomic DNA.

The target genes using cDNA of placental origin included *SLC2A1* (GLUT-1), *SLC2A3* (GLUT-3), *SLC2A4* (GLUT-4), *SLC38A4* (system A amino acid transporter isoform ATA-3), *SLC7A1* (system y+ isoform CAT-1), *SLC7A5* and *SLC7A8* (system L isoforms LAT-1 & LAT-2), and mTOR. Transcript abundance was determined by real time PCR TaqMan Gene Expression Assays (Applied Biosystems, Foster City, CA, USA) on an ABI Prism 7900HT Sequence Detector (Applied Biosystems) using cycling conditions recommended by the manufacturer. Singleplex amplification was carried out in triplicate in 384-well plates with a total reaction volume of 20 µl, containing 10 µl of TaqMan Universal PCR Master Mix (Applied Biosystems), 1 µl cDNA sample, 200 nM probe, 900 nM forward and reverse primers and 6 µl DEPC-treated water. The cDNA samples were diluted 100-fold for all genes apart from 18S (1,000-fold), mTOR (10-fold), and *SLC38A4* (undiluted). Standard curves for both the target gene and housekeeping genes, consisting of 10-fold serial dilutions (2-fold serial dilutions for *SLC38A4*) of cDNA derived from a single placental sample, were included in each plate.

Seven potential housekeeping genes (HKGs: GAPDH; beta actin; cyclophilin A; HPRT1; YWHAZ; 18s, and RPL19) were screened for stable expression. Stability of expression was evaluated by inspection of the standard deviation (SD) and coefficient of variance (CV) calculated for each HKG across a range of samples. HKGs were ordered from the most stably expressed (exhibiting the lowest variation) to the least stable one. The geometric mean of the three most stably expressed HKGs (cyclophilin A, GAPDH, and RPL19) was calculated for each sample to derive a Bestkeeper index as described by Pfaffl *et* *al* [36]. Real time PCR amplification efficiencies were calculated from the slopes of the standard curves for each target gene and HKG. Gene expression levels were expressed relative to the control or saline groups and as a ratio to HKG mRNA levels using a mathematical model; this model incorporates the efficiency of amplification for each individual transcript, thereby accounting for any variation in amplification efficiency amongst transcripts [37].

### Oligonucleotide primers and probes

Ovine sequences in GenBank for the target genes were analysed using Primer Express Software (Applied Biosystems) to determine optimum minor groove binding primer and probe locations (Table 1). A BLAST search ensured that primers and probes were not designed from homologous regions that would encode for proteins other than our target proteins. For mTOR and *SLC38A4*, ovine sequences were not available and bovine sequences were used, selecting the regions that were maximally conserved across different mammalian species.

### Protein extraction and quantification

We were unable to identify a suitable antibody against SLC2A3. For trans-membrane SLC2A1 and SLC2A4 molecules, protein was extracted on ice with 0.5 ml lysis buffer (150 mM NaCl, 50 mM Tris pH 6.8, 1 mM ethylene diamine tetraacetic acid (EDTA), 1 mM ethylene glycol tetraacetic acid (EGTA), 2 mM sodium orthovanadate). Tissue samples were homogenized for 15–20 s and centrifuged at 700 rcf for 5 min at 4°C. Supernatants were transferred to fresh tubes and pellets were re-suspended in 0.5 ml lysis buffer (2% triton X-100, 1% sodium deoxycholate and 1% sodium dodecyl sulphate, 150 mM NaCl, 50 mM Tris pH 6.8, 1 mM EDTA, 1 mM EGTA, 2 mM sodium orthovanadate). Samples were re-homogenized for 15–20 sec, left on ice for a few minutes and were then transferred back into tubes containing its supernatant. Lysates were then rotated for 30 min at 4°C. Lysates were stored at −80°C.

For mTOR, a cytosolic protein, this protocol was modified to use: 1 ml of lysis buffer (20 mM Tris (pH 7.5), 150 mM NaCl, 1 mM EDTA, 1 mM EGTA, 1% Triton X-100, Complete Mini protease inhibitor cocktail (Roche Diagnostics GmbH, Mannheim, Germany) and phosSTOP phosphatase inhibitor cocktail (Roche)). Tissue samples were then homogenized for 15–20 s and centrifuged at 16,100 rcf for 15 min at 4°C, and supernatant were transferred into fresh tubes to be stored at 80°C.

Protein content of all lysates was determined using a modified Lowry method (Bio-Rad DC-protein assay kit, Bio-Rad Laboratories, Inc, Hercules, CA, USA).

### Western blotting

Western blots for SLC2A1 and SLC2A4 were performed using pre-cast NuPAGE® 4–12% Bis-Tris Gels (Invitrogen, Carlsbad, CA, USA). The loading samples, containing protein lysate, MilliQ water, 1 M DTT (final concentration 100 mM) as a reducing agent, and 4 x NuPAGE loading dye (Invitrogen) in a total volume of 40 µl, were prepared on ice and then heated at 70°C for 10 min. 30 µl of sample containing 60 µg of protein and 8 µl lane marker (SeeBlue plus2 pre-stained standard; Invitrogen) were loaded into each well. An identical reference sample was included in each gel to account for inter-gel variability.

Gels were run at 200 V for 50 min with NuPAGE® MOPS (3-(N-morpholino) propanesulphonic acid) running buffer. 500 µl of NuPAGE antioxidants (Invitrogen) were added to the inner chamber. Proteins were transferred on to PVDF membranes using iBlotTM Gel Transfer Stack and iBlotTM Dry Blotting System (Invitrogen). Membranes were air-dried, soaked in methanol solution, rinsed with MilliQ water, and left with blocking buffer (5% non-fat dry milk, 1x PBS +0.1% Tween 20) at 4°C overnight on a shaker.

Membranes were washed with 1x PBS +0.1% Tween before incubation for 2 h at room temperature with primary antibodies (SLC2A1: dilution 1∶5,000, catalogue number ab32551; SLC2A4: 1∶2,000, ab37445; beta actin: 1∶200,000, ab6276; all Abcam plc, Cambridge, UK,). Following four 5 min washes with 1x PBS +0.1% Tween, membranes were incubated with secondary antibody (A0545, Sigma Aldrich, MO, USA 1∶10,000, for GLUTs; A9044, Sigma Aldrich 1∶200,000, for β-actin) for one hour at room temperature.

For mTOR (MW ∼289 kDa) the Western blot protocol was modified to include use of NuPage® Tris-Acetate running buffer, Hi Mark Pre-stained protein standard and NuPage® Novex 3–8% Tris-Acetate Gel (all Invitrogen) for gel electrophoresis. 80 µg total protein were added per lane and gels were run for 1 h at 150 V. Gels were then incubated in 2x NuPage® transfer buffer containing 10% methanol and 1∶1,000 antioxidant for 10 min. After transfer, membranes were blocked at room temperature for 3–4 h, then incubated overnight at 4°C with primary antibody (mTOR (#2972) and phospho-mTOR (#2971S), Cell Signaling, Danvers, MA, USA; both 1∶10,000 dilution) in PBS-Tween containing 5% bovine serum albumin.

Following incubation with secondary antibody, membranes were washed five times for 5 min with 1x PBS +0.1% Tween and then incubated for 5 min with enhanced chemiluminescence detection substrate (SuperSignal® West Dura Extended Duration Substrate kit, Pierce Biotechnology Inc, Rockford, IL, USA). Bands were visualized by exposure to autoradiographic film (AGFA, Gevaert, Belgium). Densitometric analysis was performed on a GS800 Calibrated Densitometer (Bio-Rad, Hercules, CA, USA) using the Quantity One Software (Bio-Rad), with all data normalised to the relative optical density of the beta actin band.

### Molecular weights of proteins

The SLC2A1 protein detected by SLC2A1 antibody yielded single bands of ∼61 kDa molecular weight. The specificity of antibody was tested using rat liver tissue as a positive control with an observed band of ∼30 kDa. The observed bands of SLC2A4 for sheep tissue were at ∼ 30 kDa molecular weight, for beta actin were at ∼45 kDa, and for mTOR and p-mTOR at ∼289 kDa.

### Data analysis

Blood oxygen content was calculated from measured hemoglobin, oxygen saturation, and PaO_2_ [38]. Uptakes of oxygen, glucose, and lactate were calculated for the uterus and its contents [35]. Uterine and umbilical blood flows were calculated from steady-state antipyrine diffusion [35]. Clearances of 3-O-[Me^3^H]glucose and [^14^C]urea were calculated using steady-state diffusion techniques [35]. Fetal urea production rate was calculated as the product of [^14^C]urea clearance and the (fetal artery – maternal artery) urea concentration difference [35].

Growth catheter measurements were normalized to 101 dGA, and analyzed separately for embolization (103–110 dGA) and treatment (110–131 dGA) periods. A general linear model was used with girth increment as the response variable, and treatment group and dGA as the independent variables, with dGA also included as a covariate. All other analyses used similar models except that dGA was included only as a covariate. Fetal size and organ weights were analysed by ANOVA, with Tukey's *post hoc* comparisons. SLC2A1, SLC2A4, mTOR and p-mTOR western blots were analysed using Kruskal-Wallis tests in JMP (v. 7.0; SAS institute Inc, Cary, NC, USA). All other analyses were carried out in Minitab (v.15, Pennsylvania State University, USA), with Johnson transformation as required to stabilize the variance. Data are mean±SEM or fold change (95% confidence intervals), as appropriate.

## Results

Forty-two singleton-bearing ewes (17 Control, 14 IUGR, 11 IGF1) started the experiment, and 38 (15, 12, and 11, respectively) reached the treatment phase. All data are not available for all animals due to fetal losses, catheter failures, or insufficient quantities of fetal plasma for analysis.

### Fetal growth, body and organ size

**Figure 1:**
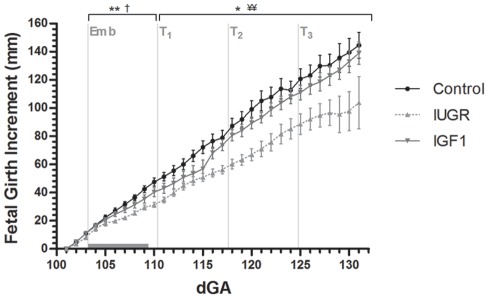
Fetal growth rate presented as the increase in girth size (mm) relative to the starting date of measurements (101 dGA). Data points are the means with SEM. *Emb* marks the start of the embolization period, which is indicated by the horizontal gray bar. At *T_1_*, *T_2_* and *T_3_* the IUGR and IGF1 groups received intra-amniotic saline or IGF-1 injections, respectively. * p<0.05 and ** p<0.01 for IUGR vs. Control; † p<0.05 for IGF1 vs. Control; ¥¥ p<0.01 for IGF1 vs. IUGR.

Fetal hind limb length and chest girth at surgery were not different amongst groups (hind limb length: Control, 17.4±1.0 mm; IUGR, 18.5±1.4 mm; IGF1, 16.5±1.5 mm; Chest girth: Control, 22.8±0.4; IUGR, 22.5±0.7 mm; IGF1, 21.4±0.6 mm). However, IGF1 fetuses had smaller biparietal diameters than IUGR fetuses (p<0.05) at surgery (Control, 49.0±0.6 mm; IUGR, 50.0±0.9 mm; IGF1 46.7±0.9 mm). Embolization reduced fetal growth rate compared with control fetuses (*p*<0.05; Fig. 1). During the treatment period, growth rate of IGF-1-treated fetuses was greater than saline-treated fetuses (*p*<0.01; Fig. 1), and not different from controls. At post mortem, saline-treated fetuses were 21% lighter, and had shorter crown-rump, limb lengths, and chest girth than controls (Table 2).

**Table 2:** Fetal body measurements at postmortem (131 dGA).

	Control	IUGR	IGF1
**Absolute Weight (g)**			
**Fetal weight**	3895±206	3087±280*	3218±261
**Adrenals**	0.5±0.1	0.6±0.1	0.6±0.2
**Brain**	44.5±1.7	41.3±2.0	42.3±2.1
**Heart**	29.3±1.3	26.7±0.2	26.1±2.5
**Kidneys**	26.3±2.0	26.8±2.6	26.4±2.2
**Liver**	145.3±14.1	123.9±16.4	112.6±10.9
**Lungs**	117.6±7.2	85.2±6.8**	99.0±4.9
**Pancreas**	3.6±0.3	3.0±0.3	3.3±0.4
**Perirenal fat**	12.6±0.8	13.7±2.8	13.1±0.8
**Spleen**	11.4±0.2	6.6±1.3*	6.5±0.6*
**Thymus**	15.1±1.9	8.0±1.9*	11.3±2.7
**Thyroid**	1.0±0.2	0.9±0.2	0.9±0.2
**Uterus**	706±33	596±57	613±60
**Placenta**	596.0±92.6	340.1±41.4*	395.0±44.8
**Placentome number**	78±5	64±5	69 ±5
**Mean placentome weight**	6.04±0.79	4.10±0.84	4.36±0.90
**Fetal: placental weight ratio**	8.04±0.77	9.28±0.77	8.51±0.87
**Relative Weight (g/kg body weight)**			
**Adrenals**	0.12±0.02	0.20±0.03*	0.18±0.02
**Brain**	11.6±0.5	13.8±0.7*	13.3±0.8
**Heart**	7.6±0.4	8.7±0.6	8.2±0.6
**Kidneys**	6.9±0.7	8.7±0.5*	8.4±0.6
**Liver**	38.0±4.1	40.4±4.3	35.5±2.5
**Lungs**	30.6±1.8	28.1±1.6	31.7±2.2
**Pancreas**	1.0±0.1	1.0±0.1	1.1±0.1
**Perirenal fat**	3.3±0.2	4.3±0.4*	4.2±0.3*
**Spleen**	3.0±0.5	2.0±0.2*	2.0±0.1
**Thymus**	3.8±0.3	2.4±0.4*	3.3±0.6
**Thyroid**	0.3±0.1	0.3±0.1	0.3±0.1
**Length (cm)**			
**CRL**	43.9±1.0	40.6±1.0*	41.5±1.2
**BPD**	60.9±1.3	60.4±1.5	58.4±1.4
**Chest (girth)**	33.6±0.6	31.1±0.9*	31.6±0.8
**Forelimb Hock-hoof**	15.1±0.5	13.7±0.4*	14.0±0.6
**Forelimb**	27.8±0.4	25.6±0.7*	26.6±0.8*
**Hindlimb Hock-hoof**	17.9±0.3	16.5±0.3*	16.5±0.7*
**Hindlimb**	32.9±0.4	29.8±0.7**	30.5±1.2*

Values are mean±SEM. *p<0.05, **p<0.01 for comparison with Control. Control (*n* = 11), IUGR (*n* = 9), and IGF1 (*n* = 7).

Total placental weight was reduced in IUGR fetuses compared with controls (Table 2); IGF1 placental weight was not significantly different from either Control or Saline. Both IUGR and IGF1 animals tended to have decreased placentome number and average placentome weight compared with controls, but these differences were not statistically significant (Table 2). Absolute fetal lung, thymus, and spleen weights were also markedly reduced in saline-treated fetuses; thymus and spleen weights remained reduced when corrected for body weight. In contrast, relative brain, kidney, adrenal, and peri-renal fat weights were increased compared with controls (Table 2). IGF-1-treated fetuses were not significantly different in weight, length, or chest girth from either the Control or saline-treated fetuses. However, IGF1 fetuses had shorter limbs and lighter spleens than controls, as well as greater relative peri-renal fat mass (Table 2).

### Blood gas and metabolite concentrations

**Figure 2:**
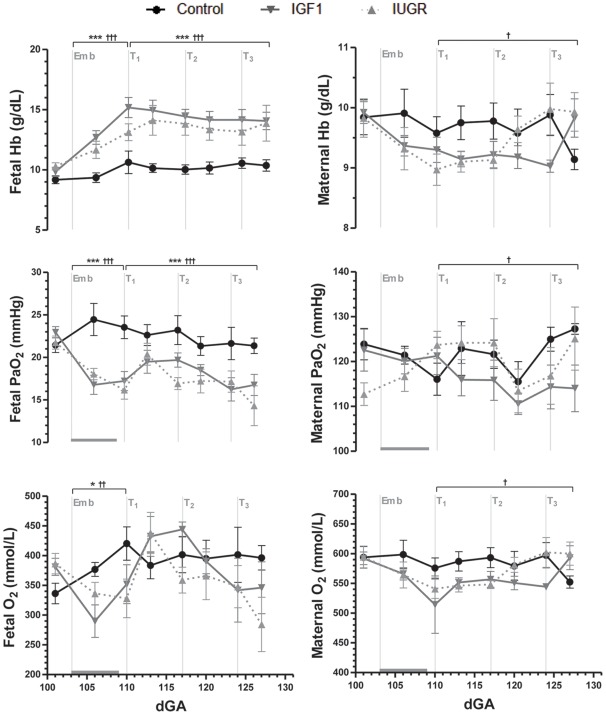
Fetal and maternal arterial oxygen and hemoglobin concentrations. Data are means with SEM. *Emb* marks the start of the embolization period, which is indicated by the horizontal gray bar. At *T_1_*, *T_2_* and *T_3_* the IUGR and IGF1 groups received either a saline or IGF-1 intra-amniotic injection, respectively. Hb  =  hemoglobin. ^*^ *p*<0.05 and ^***^ *p*<0.001 for IUGR vs. Control; ^†^ *p*<0.05, ^††^ *p*<0.01 and ^†††^ *p*<0.001 for IGF1 vs. Control.

**Figure 3:**
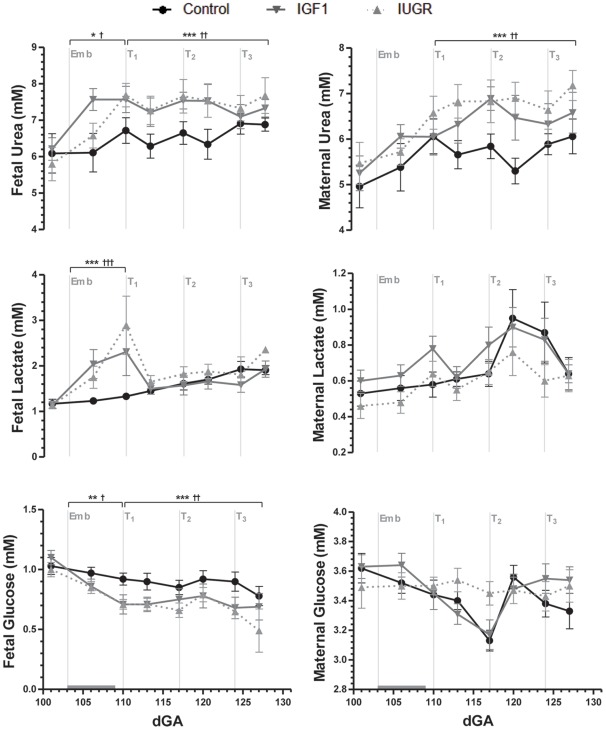
Fetal and maternal plasma metabolite concentrations. Data are means with SEM. *Emb* marks the start of the embolization period, which is indicated by the horizontal gray bar. At *T_1_*, *T_2_* and *T_3_* the IUGR and IGF1 groups received either a saline or IGF-1 intra-amniotic injection, respectively. ^*^ *p*<0.05, ^**^ *p*<0.01 and ^***^ *p*<0.001 for IUGR vs. Control; ^†^ *p*<0.05, ^††^ *p*<0.01 and ^†††^ *p*<0.001 for IGF1 vs. Control.

Embolization reduced fetal PaO_2_ and O_2_ content (Fig. 2), SaO_2_ (data not shown; *p*<0.001), and increased fetal hemoglobin concentration compared with controls (Fig. 2). Fetal pH was unaffected by embolization (data not shown), despite significantly increased fetal plasma lactate concentrations (Fig. 3). Embolization decreased fetal plasma concentrations of glucose (Fig. 3) and the non-essential urea cycle amino acids arginine and ornithine (Table 3). Fetal plasma concentrations of the essential amino acids were not altered by embolization, but urea concentrations were increased (*p*<0.05; Fig. 3) and taurine concentrations approximately doubled (Table 3).

**Table 3:** Fetal plasma concentrations of essential and non-essential amino acids during the embolization and treatment periods.

	Embolization			Treatment		
	Control (*n* = 10)	IUGR (*n* = 6)	IGF1 (*n* = 9)	Control (*n* = 5)	IUGR (*n* = 4)	IGF1 (*n* = 6)
**ESSENTIAL**						
**Isoleucine**	98.1±8.6	104.9±8.9	83.8±7.6	86.6±6.1	94.5±6.3	76.7±3.8
**Leucine**	157.8±10.3	168.1±13.3	137.4±9.7	134.6±8.6	159.6±12.9	130.8±5.5
**Lysine**	98.9±7.9	91.9±26.7	92.2±7.0	89.6±11.0	85.5±8.0	89.3±6.9
**Methionine**	26.2±2.8	30.0±4.3	30.2±3.6	23.0±2.0	30.2±3.9	25.1±2.8
**Phenylanine**	81.4±7.3	86.9±8.7	94.2±7.3	80.6±5.8	87.7±5.1	81.2±4.4
**Threonine**	319.3±28.8	245.7±25.3	264.8±38.6	329.7±35.0	266.2±18.7	256.2±34.4
**Valine**	435.5±28.4	468.8±49.8	374.3±33.3	385.0±21.9	462.0±26.7	374.5±18.1†
**BCAA**	691.4±45.5	741.8±68.1	565.9±43.7	606.2±35.7	716.1±44.6	582.1±26.4†
**NON-ESSENTIAL**						
**1-methylhistidine**	3.5±0.5	2.1±0.4	2.7±0.3	3.7±0.4	3.6±0.7	3.6±0.6
**3-methylhistidine**	32.3±5.3	24.6±3.4	25.2±4.2	30.8±5.2	41.9±6.1	30.3±5.1
**Alanine**	227.2±12.6	267.9±24.1	239.5±17.8	232.2±13.3	291.2±18.4	252.9±21.5
**Arginine**	83.9±5.0	52.0±4.6**	62.7±5.2*	79.6±6.4	68.2±5.7	74.9±4.0
**Asparagine**	50.7±4.2	52.6±5.5	49.1±4.1	42.2±2.7	54.6±4.0*	43.6±3.0
**Aspartate**	7.1±0.7	7.6±1.4	6.0±0.6	6.5±0.5	7.9±0.9	5.8±0.4†
**Citrulline**	193.7±13.1	157.7±8.0	178.1±14.2	182.5±13.2	169.8±6.2	212.6±14.3
**Glutamine**	405.9±25.0	410.9±32.4	401.1±34.8	363.0±19.3	448.6±26.5	380.9±27.3
**Glutamate**	45.1±3.9	35.8±7.5	32.2±3.1	42.0±3.4	41.8±4.6	38.4±3.4
**Glycine**	305.1±29.5	293.3±12.8	269.5±14.3	279.4±22.0	294.4±14.3	265.7±18.9
**Histidine**	25.0±2.1	25.1±3.5	26.3±2.0	22.6±1.8	34.7±2.9**	25.8±2.4†
**Hydroxyproline**	44.4±3.9	45.6±3.6	44.4±3.2	44.3±2.6	54.7±4.1	47.3±3.0
**Ornithine**	71.6±6.1	44.5±4.9**	50.1±4.5*	60.5±4.0	52.0±4.0	57.3±5.2
**Proline**	147.3±12.1	193.5±24.8	173.7±24.3	134.2±10.8	164.6±4.6	156.2±10.2
**Serine**	422.3±49.1	488.4±61.8	400.4±57.0	424.9±51.6	575.1±55.1	437.3±51.2
**Taurine**	90.7±18.0	201.0±31.6*	175.5±29.9*	86.4±16.4	160.6±33.5	236.0±103.0
**Tyrosine**	108.6±12.5	90.7±13.1	137.3±25.7	113.5±9.2	83.3±5.4	121.3±8.8†

Values are mean±SEM (µMol/L). *p<0.05 and **p<0.01 for comparison with Control, while †*p*<0.05 for comparison with IUGR.

There were no significant differences in maternal blood gas variables or metabolites amongst groups during embolization (Fig. 2, 3). However, maternal hemoglobin concentration (*p*<0.05), PaO_2_ (*p*<0.05) and O_2_ content (*p*<0.05) were lower in IGF1 group ewes than controls, and not significantly different from saline-treated ewes (Fig. 2).

IGF-1 treatment had no significant effect on fetal blood gas and metabolite concentration (Figs. 2, 3). In both embolized groups, fetal plasma urea concentrations remained elevated (*p*<0.01) and glucose concentrations depressed (*p*<0.01; Fig. 3). IGF-1 treatment decreased fetal plasma concentrations of aspartate, histidine and the branched-chain amino acids compared with saline-treated fetuses, and increased tyrosine concentrations (Table 3).

IGF-1 treatment had no effect on maternal plasma metabolite concentrations. However, maternal urea concentrations remained significantly higher in both embolized groups compared with controls (*p*<0.01; Fig. 3).

### Placental function

Embolization considerably reduced uterine blood flow (Control = 1544±131, IUGR = 950±161, IGF1 = 947±107 ml/min•kg; *p*<0.05). Uterine uptake of glucose was significantly reduced in saline fetuses compared with controls; IGF1 fetuses were not significantly different from controls or saline fetuses (Control = 388±38, IUGR = 249±28, IGF1 = 290±32 μmol/min; *p*<0.05). Embolization did not change clearance of 3-O-[Me^3^H]glucose (Control = 392±185, IUGR = 142±40, IGF1 = 182±57 ml/min•kg; *p* = 0.30) or urea (Control = 50±83, IUGR = 98±21, IGF1 = 161±44 ml/min•kg; *p* = 0.48), and did not change uterine uptakes of lactate and oxygen (data not shown).

### Hormones

**Figure 4:**
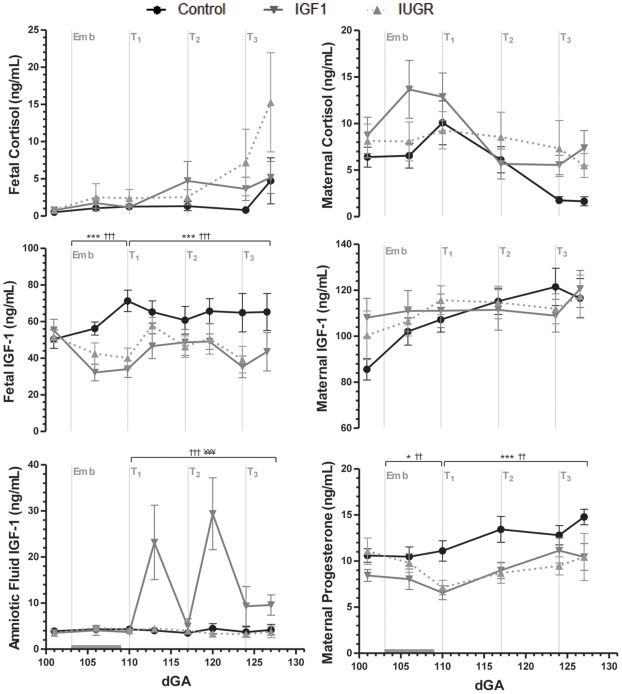
Hormone concentrations in fetal plasma, maternal plasma and amniotic fluid. Data are means with SEM. *Emb* marks the start of the embolization period, which is indicated by the horizontal gray bar. At *T_1_*, *T_2_* and *T_3_* the IUGR and IGF1 groups received either a saline or IGF-1 intra-amniotic injection, respectively. ^*^ *p*<0.05 and ^***^ *p*<0.001 for IUGR vs. Control; ^††^ *p*<0.01 and ^†††^ *p*<0.001 for IGF1 vs. Control; ^¥¥¥^ *p*<0.001 for IGF1 vs. IUGR.

Embolization had no significant effect on IGF-1 concentrations in maternal plasma or amniotic fluid, but reduced concentrations in fetal plasma (*p*<0.001; Fig. 4). Amniotic fluid concentrations of IGF-1 increased approximately 5-fold by three days after the first IGF-1 injection (*p*<0.001), but were no longer different from control and saline groups by the time of the second injection. Thereafter, amniotic fluid IGF-1 concentrations remained higher in IGF-1-treated animals (Fig. 4).

Fetal plasma IGF-1 concentrations remained significantly lower than controls in both embolized groups during the treatment period (*p*<0.001), and were unaffected by IGF-1 treatment (Fig. 4).

Maternal and fetal plasma cortisol concentrations were not significantly different amongst groups throughout the experiment (Fig. 4). However, embolization decreased maternal progesterone concentrations (*p<*0.05), and these remained lower in both embolized groups than in control ewes (*p*<0.01) throughout the treatment period (Fig. 4).

### Placental nutrient transporters

**Figure 5:**
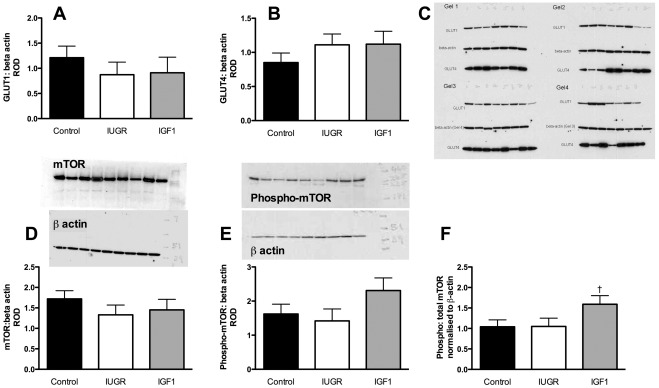
Placental protein levels of glucose transporters 1 and 4 and mammalian target of rapamycin (mTOR). [A] SLC2A1 (GLUT1), [B] SLC2A4 (GLUT4), [C] total mTOR, [D] phosphorylated mTOR (phospho-mTOR), and [E] the ratio of phospho-mTOR to total mTOR. Data are relative optical densities (ROD, mean±SEM) of the protein of interest normalised to the ROD of the loading control (beta-actin). Black bar, control, *n* = 11; white bar, IUGR, *n* = 9; grey bar, IGF1, *n* = 7. Complete gels are shown, with all four gels for SLC2A1 and SLC2A4 and representative gels for mTOR and phospho mTOR. ^†^ *p* = 0.06 compared with IUGR.

Embolization decreased placental mRNA levels of *SLC2A1* compared with controls, but had no effect on *SLC2A3* or *SLC2A4* mRNA levels (Table 4). IGF-1 treatment increased *SLC2A1* and *SLC2A4* mRNA levels compared with saline treatment (Table 4), but did not affect *SLC2A3*. Placental SLC2A1 and SLC2A4 protein levels were not significantly different amongst groups (Fig. 5). Embolization decreased placental mRNA levels of *SLC7A1* and *SLC7A8* by 31 and 38%, respectively, but had no effect on *SLC38A4* or *SLC7A5* (Table 4). IGF-1 treatment increased mRNA levels of placental *SLC7A1* by 57%, *SLC7A8* by 32%, and *SLC38A4* by 500% compared with saline treatment (Table 4). Thus, *SLC38A4* mRNA levels were 5-fold higher in IGF1 fetuses than in controls, although *SLC7A1* mRNA levels were not significantly different from controls (Table 4). *SLC7A5* mRNA levels were not altered by embolization or IGF-1 treatment (Table 4).

**Table 4:** mRNA levels for candidate genes in the placenta.

	Relative Expression Ratio (95% CI)		
	IUGR: Control	IGF1: Control	IGF1: IUGR
**System A:** ***SLC38A4***	1.06 (0.65–1.71)	5.30 (2.77–10.1)*	5.0 (2.35–10.7)*
**System y+:** ***SLC7A1***	0.62 (0.58–0.66)*	0.96 (0.61–1.52)	1.57 (1.07–2.28)*
**System L:** ***SLC7A5***	1.06 (0.82–1.38)	0.92 (0.47–1.80)	0.87 (0.41–1.82)
**System L:** ***SLC7A8***	0.69 (0.62–0.77)*	0.91 (0.68–1.21)	1.32 (1.11–1.57)*
***SLC2A1*** ** (GLUT1)**	0.67 (0.62–0.71)*	1.03 (0.69–1.43)	1.50 (1.12–2.01)*
***SLC2A3*** ** (GLUT3)**	1.01 (0.85–1.17)	1.06 (0.69–1.51)	1.03 (0.72–1.46)
***SLC2A4*** ** (GLUT4)**	0.84 (0.63–1.12)	1.12 (0.93–1.35)	1.33 (1.02–1.74)*
**mTOR**	0.96 (0.85–1.08)	1.27 (0.94–1.70)	1.32 (1.00–1.75)^†^

Data are fold change (95% CI) relative to the control or IUGR group expressed as a ratio to the Bestkeeper index derived from three housekeeping genes (cyclophilin A, GAPDH and RPL19) [36], [37]. First column: IUGR group compared with control; middle column: IGF1 group compared with Control; last column: IGF1 group compared with IUGR. Where 95% confidence intervals do not cross 1 the two groups are significantly different at the 5% level (shown by *, with ^†^representing *p* = 0.05). Control (*n* = 11), IUGR (*n* = 9), and IGF1 (*n* = 7).

### Placental mTOR

Embolization did not alter mRNA levels of mTOR (Table 4) or protein levels of mTOR or phospho-mTOR compared with controls (Fig. 5). IGF-1 treatment tended to increase mTOR mRNA levels compared to IUGR (*p* = 0.05 Table 4), but did not change protein levels of total mTOR or its phosphorylated form (p-mTOR; Fig. 5). The ratio of p-mTOR to total mTOR was 1.5-fold greater in placentae from IGF-1-treated fetuses compared with saline-treated IUGR fetuses (*p* = 0.06; Fig 5).

## Discussion

This study demonstrates that a once-weekly intra-amniotic injection of a low dose of IGF-1 increases growth of ovine fetuses with growth-restriction secondary to placental insufficiency. This increased fetal growth is likely to be, at least in part, due to increased expression of placental amino acid transporters mediated by mTOR. These findings suggest a potential approach to intrauterine treatment of fetal growth restriction that may be clinically acceptable.

Fetal plasma IGF-1 concentrations were unaltered with weekly intra-amniotic IGF-1 treatment, consistent with findings following thrice-weekly treatment with 120 µg IGF-1 [18]. In contrast, daily administration of IGF-1 (20 µg/dose) for ten days significantly reduced fetal plasma IGF-1 concentrations [19]. These consistent findings of unchanged or decreased plasma IGF-1 concentrations with a variety of intra-amniotic treatment regimens suggests the increased fetal growth rate following intra-amniotic IGF-1 treatment was not due to direct growth-promoting effects of IGF-1, even though we have demonstrated previously that IGF-1 given into amniotic fluid is taken up intact across the fetal gut and into the systemic circulation [17].

It is possible, however, that intra-amniotic IGF-1 stimulates fetal nutrient uptake from amniotic fluid. Human studies suggest that fetuses may obtain up to 15% of daily nitrogen requirements from swallowed amniotic fluid [39], [40]. We have previously demonstrated that daily doses of intra-amniotic IGF-1 are swallowed by the fetus [17], increase glutamine utilization by the fetal gut [34], and restore the delayed gut development seen in IUGR fetuses [19]. However, when we attempted to maximise the growth-promoting effects of intra-amniotic IGF-1 by co-administering additional nutrients to amniotic fluid, the effect of IGF-1 was abrogated [18], suggesting that increased nutrient uptake across the fetal gut is unlikely to be the mechanism behind the observed increase in fetal growth.

In IUGR secondary to placental insufficiency, the primary problem is impaired fetal nutrient supply. In sheep, the acute responses to a reduced supply of oxidative substrates are mobilization of endogenous substrates and inhibition of protein accretion. Amino acids from the carcass are mobilized, and protein oxidation may account for up to 80% of oxygen consumption in IUGR fetuses [41]. If the impaired nutrient supply persists, amino acids appear to substitute glucose as the major oxidative fuel, resulting in a 50% or greater increase in fetal urea production [41].

The significantly increased fetal plasma urea concentrations in the current study are consistent with this response. The amino acids utilized by the fetus could arise either from increased transport across the placenta, or from breakdown of fetal tissue. Our finding of increased plasma concentrations of branched-chain amino acids (BCAA) in IUGR fetuses is consistent with muscle breakdown. In the placental specific *igf2* knockout mouse, a compensatory increase in amino acid and glucose transport across the placenta occurs prior to the onset of IUGR. Failure of this compensation appears to precede the onset of fetal growth restriction [42].

Others have also suggested that reduced placental amino acid transport may precede the onset of IUGR [25], and have reported down-regulation of amino acid transporter activities in IUGR placentae [43]. Consistent with these data, we found decreased *SLC7A1* and *SLC7A8* mRNA levels in IUGR placentae. However, *SLC38A4* and *SLC7A5* mRNA levels were not altered, which may reflect different timings in the failure of compensation for different amino acid transporters. Alternatively, these mRNA levels reflect expression in whole placentomes, meaning that differential regulation of amino acid transporters on the maternal and fetal-facing membranes may be masked. Further, amino acid transporter activity may also be altered without changes in gene expression due to post-translational or conformational changes [44].

An alternative possible mechanism for the growth-promoting effects of IGF-1 in this study may be effects on the placenta, via either increased blood flow or increased placental nutrient transport. IGF-1 has vasodilatory effects that are mediated by nitric oxide [45]. However, attempts to improve uterine artery blood flow by administration of nitric oxide donors to the mother have not been effective [13], [14], and we did not find an effect of IGF-1 treatment on uterine blood flow. Although we have previously demonstrated that a continuous intravenous infusion of a similar daily dose of IGF-1 to the fetus alters placental clearance of glucose and amino acid analogues [19], [35], there was no effect of intra-amniotic IGF-1 on glucose uptake across the placenta in this study.

However, weekly intra-amniotic IGF-1 treatment did result in significant up-regulation of mRNA levels of placental transporters for neutral (*SLC38A4*), cationic (*SLC7A1*), and branched-chain amino acids (*SLC7A8*), which could provide a mechanism for increased substrate supply to the IUGR fetus. To our knowledge *SLC38A4* has not been studied in the sheep placenta before, although expression in a variety of bovine tissues has been reported [46]. It is an isoform of system A found in both the basal and microvillous membranes of the human placenta [47], and is also found in the bovine placental caruncle [46]. *SLC38A4* transports neutral amino acids with small side chains (e.g. alanine, glycine, and glutamine) in a sodium- and pH-dependent manner, and cationic amino acids (e.g. arginine and lysine) in a sodium- and pH-independent manner. The system L transporter (*SLC7A5* and *SLC7A8*) carries neutral amino acids with large and branched side chains, such as leucine, isoleucine, and phenylalanine [48]. *SLC7A1*, an isoform of system y+, the major cationic amino acid transporter in the placenta [48], transports amino acids such as lysine and arginine. Our data demonstrate considerable up-regulation (between 132 and 500%) of isoforms of all three amino acid transporter systems studied, suggesting that effects of IGF-1 on the placental may explain the increased fetal growth seen. Functional studies of amino acid uptake are required to verify this. IGF-1 has been reported to increase placental system A activity and expression in cultured human trophoblast cells and in BeWo choriocarcinoma cell lines [26], [49]. The role of IGF-1 in regulation of system y+ transporter expression is poorly understood, but may be similar to that described for system A.

Increased transplacental transport of other amino acids, mediated by the increased levels of *SLC38A4, SLC7A1,* and *SLC7A8*, may have decreased the need for fetal muscle breakdown to provide an alternative oxidative substrate [50], thereby reducing placental glucose metabolism and conserving other metabolic fuels for the fetus [34]. This is supported by the observed decrease in plasma BCAA concentrations in IGF-1 treated fetuses.

Activity of the placental amino acid transporters in the human placenta is mediated by mTOR [25], [51]. Upstream regulators of mTOR activity in trophoblast cells include glucose concentrations, BCAA concentrations, insulin, and IGF-1 [51], [52]. mTOR is activated by phosphorylation at Ser^2448^, and placental expression of p-mTOR may be a more reliable indicator of mTOR activity [53]. Placental total mTOR protein has been reported to be decreased in human IUGR [54], [55] and in rat pregnancies subjected to protein undernutrition [56], although it has also been reported to increase in an ovine paradigm of IUGR [57]. There are few data on the interactions between the mTOR and IGF-AKT signaling pathways in the placenta. However, in other tissues, the close integration of these pathways with the p53 pathway in the sensing of nutrient and growth factor availability is well described, as well as the subsequent transduction of these signals into cell growth and proliferation [58]. Nonetheless, in human trophoblast cells, the up-regulation of system A activity by IGF-1 has been shown to be mediated via increased mTOR activity [51]. We found that mRNA and protein levels of both total mTOR and p-mTOR were unchanged in IUGR fetuses. IGF-1 treatment resulted in an increase in mRNA levels of mTOR of borderline statistical significance (*p* = 0.05), with a p-mTOR:mTOR ratio that tended to be greater than in saline-treated IUGR fetuses (*p* = 0.06). Although our data on the effect of IGF-1 on mTOR are not conclusive, they do suggest that further investigation of the role of the mTOR pathway in mediating the effects we report is warranted.

Embolization and IUGR also decreased mRNA levels of *SLC2A1* in IUGR placentae, consistent with the decreased uterine glucose uptake that we report. However, the decreased *SLC2A1* mRNA levels were not matched by a significant up-regulation in GLUT-1 protein expression, presumably due to inadequate sensitivity of the Western blotting technique to post-translational changes [59], or to the fact that we measured protein levels of membrane-bound GLUT-1 that may not reflect total protein in the cell. We could not study placental GLUT-3 expression because of the unavailability of a suitable antibody, but we provide the first evidence for the presence of GLUT-4 in the ovine placenta. GLUT-4 has been reported in rodent and human placentae, predominantly in early gestation [22], [23] but also at term [60], [61]. There are few data on GLUT-4 regulation in response to an adverse fetal nutritional environment, but levels were not reported to change in rabbit pregnancies complicated by maternal hypercholesterolemia [61], or in a small number of human term placentae from pregnancies complicated by IUGR or maternal diabetes [60].

The effect of IGF-1 on placental GLUT expression in IUGR placentae has not been reported previously. We found that IGF-1 treatment of IUGR fetuses increased placental *SLC2A1* and *SLC2A4*, but not *SLC2A3* mRNA levels, and also did not change placental GLUT-1 and GLUT-4 protein levels. Further, IGF-1 treatment did not affect fetal blood glucose concentrations or placental glucose uptake, although the decreased uterine uptake of glucose seen in IUGR animals was partially reversed with IGF-1 treatment. Thus, increase in mRNA levels of *SLC2A1* and *SLC2A4* with IGF-1 treatment may not reflect similar changes in activity.

In conclusion, this study demonstrates that weekly intra-amniotic IGF-1 injections increase fetal growth trajectory without apparent adverse effects on the fetus. In particular, fetal blood oxygen content is maintained. Furthermore, IGF-1 treatment up-regulates mRNA levels of placental transporters for neutral, cationic, and branched-chain amino acids, possibly via increased activation of the mTOR pathway. This may be a mechanism for increased substrate supply to IUGR fetuses, explaining the observed increase in fetal growth. A once-weekly intrauterine therapy for the IUGR fetus could be of clinical utility if the benefits outweighed the risks. However, although we have reproducibly demonstrated a positive effect of intra-amniotic IGF-1 treatment in growth-restricted fetal sheep, there are no data on postnatal outcomes, either short-term or long-term. Future studies should address critical postnatal outcomes such as perinatal morbidity and mortality, as well as long-term outcomes, including somatotrophic axis function and body composition.
